# Supplementary material for: Phytochemistry, Ethnopharmacology, Pharmacokinetics and Toxicology of Cnidium monnieri (L.) Cusson
Source: Int J Mol Sci. 2020 Feb 3;21(3):1006. doi: 10.3390/ijms21031006 (PMC7037677; doi:10.3390/ijms21031006)
Supplement: Supplementary file 1 [file ijms-21-01006-s001.pdf]

Supplementary Table 1. Chemical compounds isolated from Cnidium monnieri (L.) Cusson

Name	Pubchem ID or chemspider ID	Extraction methods, references, voucher numbers or specimens
Coumarins		
2′-Acetylangelicin	PubChem SID: 52466591	Extraction method: CEC Reference: [1]
Bergapten	PubChem CID: 2355	
Imperatorin	PubChem CID: 10212	
Osthole	PubChem CID: 10228	
Isoimperatorin	PubChem CID: 68081	Extraction method: HPLC<br>Reference: [2]
Isopimpinellin	PubChem CID: 68079	
Xanthotoxin	PubChem CID: 4114	
Columbianetin	PubChem CID: 92201	Extraction method: GC-MS<br>Reference: [3]<br>(with voucher specimen)
Isoporalen<br>Synonyms: Angelicin	PubChem CID: 10658	
Fraxetin	PubChem CID: 5273569	Extraction method: UHPLC-ESI-Q-TOF-MS<br>Reference: [4]
6,7,8-Trimethoxycoumarin	PubChem CID: 3083928	
Murrayacarpin A	ChemSpider ID: 901688	
Limettin	PubChem CID: 2775	
Scoparone	PubChem CID: 8417	
Isofraxidin	PubChem CID: 5318565	
7-Methoxy-8-formylcoumarin	PubChem SID: 272958589	

2′-Deoxymeranzin hydrate	PubChem CID: 10659145	
Oxypeucedanin hydrate	PubChem CID: 17536	
Peroxymurraol	No results on PubChem but chemical structure presented in previous review	
Peroxyauraptenol	No results on PubChem but chemical structure presented in previous review	
Auraptenol	PubChem CID: 13343541	
Micromarin-F	PubChem CID: 10730037	
Demethyl-auraptenol	No results on PubChem but chemical structure presented in previous review	
Albiflorin-3	PubChem SID: 274183832	
Osthenol	PubChem CID: 5320318	
7-Demethylsuberosin	PubChem CID: 5316525	
Cnidilin	PubChem CID: 821449	
Angenomalin	PubChem CID: 51520704	
Meranzin hydrate	PubChem CID: 5070783	Extraction method: HPLC-2D-CCC<br>Reference: [5]<br>(Voucher number: 20130405-SCZ)
Cnidimoside A	PubChem CID: 21603474	
Kaempferol	PubChem CID: 5280863	Extraction method: HPLC–ESI-MS<br>Reference: [6]<br>(with voucher specimen)
Isorhamnetin	PubChem CID: 5281654	
Quercitrin	PubChem CID: 5280459	
Luteolin	PubChem CID: 5280445	

Quercetin	PubChem CID: 5280343	
Hesperidin	PubChem CID: 10621	
Rutin	PubChem CID: 5280805	
Psoralen	PubChem CID: 6199	Extraction method: HFCF + HPLC<br>Reference: [7]
Oxypeucedanin	PubChem CID: 160544	
Cnidimol D	PubChem CID: 70682746	Extraction method: HPLC-PDA-ESI-ITMS^n/TOF-MS<br>Reference: [8]
2′-Hydrate-deoxymeranin	No results on PubChem but chemical structure presented in previous review	
Phlojodicarpin	No results	
(E/Z) 7-methoxy-8-(3-methylbuta1,3-dien-1-yl)-2H-chromen-2-one	No results	
Karenin	PubChem CID: 6440661	
Columbianadin	PubChem CID: 6436246	
Cniforin A	PubChem SID: 274303750	
Daucosterol	PubChem CID: 5742590	Extraction method: SGCC<br>Reference: [9]<br>Voucher number: PHD20090318
Xanthotol	PubChem CID: 65090	Extraction method: UPLC/ESI-TOF MS/MS<br>Reference: [10]
O-isovalery columbianetin	No results on PubChem but chemical structure presented in previous review	Extraction method: N/A<br>Reference: [11]
Iisogosferol	No results on PubChem but chemical structure presented in pr	Extraction method: N/A

	evious review	Reference: [12]
Archangelicin	PubChem CID: 5281371	
(3′R)-3′-hydroxycolumbianetin	No results on PubChem but chemical structure presented in previous review	
Cniforin B	No results on PubChem but chemical structure presented in previous review	
6-methoxy-8-methylcoumafin	No results on PubChem but chemical structure presented in previous review	Extraction method: TLC-MS<br>Reference: [13]
Cnidimonal	PubChem CID: 10597338	Extraction method: HPLC + GC-MS<br>Reference: [14]<br>(Voucher number: TMPW19080 in Japan and C-2103 in China)
Cnidimarin	PubChem CID: 101027478	
1′-O-β-D-glucopyranosyl (2R,3S)-3hydroxynodakenetin	No results on PubChem but chemical structure presented in previous review	Extraction method: $^{1}H+^{13}C$-NMR,<br>IR + UV spectroscopy<br>SHPLC + CC + TLC<br>Reference: [15]
7-O-methylphellodenol-B	No results on PubChem but chemical structure presented in previous review	Extraction method: $^{1}H+^{13}C$-NMR<br>UV spectroscopy<br>CC + TLC + SPHPLC<br>Reference: [16]
7-Methoxy-8-(3-methyl-2,3-epoxy-1-oxobutyl)<br>chromen-2-one	PubChem CID: 132277605	
3′-O-methylvaginol	No results on PubChem but chemical structure presented in previous review	

Hassanon	No results on PubChem but chemical structure presented in previous review	
E-murraol	No results on PubChem but chemical structure presented in previous review	
Z-murraol<br><br>Synonyms: Murraol	PubChem CID: 15593213	
3′-O-methylmurraol	PubChem CID: 137796425	Extraction method: $^{1}H+^{13}C$-NMR<br>IR + UV spectroscopy<br>ESI + HRESI<br>CC + TLC + PTLC<br>Reference: [17]<br>(Voucher number: CM-201010)
Rel-(1′S,2′S)-1′-O-methylphlojodicarpin	No results on PubChem but chemical structure presented in previous review	
(1′S,2′S)-1′-O-methylvaginol	No results on PubChem but chemical structure presented in previous review	
Isobergapten	PubChem CID: 68082	Extraction method: ESI-MS<br>Reference: [18]<br>(Voucher number: N20140901)
volatile constituents		
α-Pinene<br>Synonyms: L-Pinene	PubChem CID: 6654	Extraction method: GC-MS<br>Reference: [19]
Camphene	PubChem CID: 6616	
Limonene	PubChem CID: 22311	

Bornyl acetate	PubChem CID: 6448	
2-Phenyl-2-(phenylmethyl)-1,3-Dioxolane	No results	
Cyclobutanol	PubChem CID: 76218	
Pyruvic acid	PubChem CID: 1060	
8-Hexyl-8-pentyl-hexadecane	PubChem CID: 521602	
1,2,4-Benzenetricarboxylic acid-1,2-dimethyl ester	PubChem CID: 610016	
2-[4-(1,1-Dimethylethyl) phenoxy]-propanoic acid	No results	
2-Methyl-docosane	PubChem CID: 519145	
O-Acetylcolumbianetin	PubChem CID: 161409	Extraction method: PCEC<br>Reference: [20]
Isopropyl-3-methylbutanoate	PubChem CID: 61914	Extraction method: GC-MS<br>Reference: [21]
Tricyclene	PubChem CID: 79035	
β-Pinene	PubChem CID: 14896	
Myrcene<br>Synonyms: β-Myrcene	PubChem CID: 31253	
α-Terpinene	PubChem CID: 7462	
1, 3, 7-Octatirene, 3-7-dimethyl	PubChem CID: 5320249	
β-ocimene<br>Synonyms: Trans-ocimene	PubChem CID: 5281553	
γ-terpinene	PubChem CID: 7461	

Cis sabinene hydrate	PubChem CID: 12315152
Terpinolene	PubChem CID: 11463
1,3,8-p-Menthatriene<br><br>Synonyms: 1,3,8-para-Menthatriene	PubChem CID: 176983
Sabinene hydrate	PubChem CID: 62367
Linalool oxide	PubChem CID: 102611
p-Mentha-1,5,8-triene	PubChem CID: 527424
Trans-p-2,8-menthadien-1-ol	PubChem CID: 12618691
Neo-allo-ocimene	PubChem CID: 5371125
Camphor<br><br>Synonyms: 1, 7, 7-Trimethyl-bicyclo [2.2.1] heptan-2-one	PubChem CID: 2537
P-Mentha-2,8-dien-1-ol<br><br>Synonyms: 1-Methyl-4-(1-methylethenyl)-2-cyclohexen-1-ol	PubChem CID: 155626
L-Borneol<br><br>Synonyms: Borneol (L)	PubChem CID: 1201518
3-Cyclohexen-1-ol, 4-methyl-1-(1-methylethyl)<br><br>Synonyms: Terpinene-4-ol; 4-Terpinenol; 4-Carvomenthenol; 4-Methyl-1-(1-methylethyl)-3- cyclohexen-1-ol	PubChem CID: 11230
cis-Dihydrocarvone	PubChem CID: 443167
Bicyclo [5.1.0] octane, 8-(1-methylethylidene)	PubChem CID: 535068
α-Fenchyl acetate<br><br>Synonyms: Fenchyl acetate;	PubChem CID: 107217
Perillyl alcohol	PubChem CID: 10819

Synonyms: Perilla alcohol		
2, 6-Octadienoic acid, 3, 7-dimethyl-, methyl ester<br><br>Synonyms: Methyl geranate; 2,6-Octadienoic acid,3,7-dimethyl-, methyl ester	PubChem CID: 5365910	
Carvyl acetate<br><br>Synonyms: 2-Cyclohexen-1-ol, 2-methyl-5-(1-methylethenyl)-, acetate	PubChem CID: 7335	
Neryl acetate	PubChem CID: 1549025	
α-Copaene	PubChem CID: 70678558	
β-Bourbonene	PubChem CID: 62566	
β-Farnesene<br><br>Synonyms: Trans-β-Farnesene; 7,11-Dimethyl -3-methylene-1,6,10-dodecatriene	PubChem CID: 5281517	
Germacrene D	PubChem CID: 5317570	
β-Bisabolene	PubChem CID: 10104370	
δ-cadinene	PubChem CID: 12306054	
Caryophyllene oxide	PubChem CID: 1742210	
Geranyl pentanoate	PubChem CID: 6436375	
Cis-asarone<br><br>Synonyms: β-Asarone	PubChem CID: 5281758	
2-Pentadecanone,6,10,14-trimethyl<br><br>Synonyms: 6, 10, 14-Trimethyl-2-pentadecan one	PubChem CID: 10408	
3-Carene<br><br>Synonyms: 3,7,7-Trimethyl-Bicyclo [4.1.0] he pt-3-ene	PubChem CID: 26049	Extraction method: GC-MS<br><br>Reference: [22]

D-Limonene	PubChem CID: 440917
1-Methyl-4-(1-methylethyl)-cyclohexene	PubChem CID: 21671
2, 6-Dimethyl-2, 4, 6-octatriene	PubChem CID: 5368821
2-Isopropenyl-3-methylene-cyclohexanol	PubChem CID: 564552
2-Methyl-5-isopropenyl-2-enyl ester acetic acid	No results
Neryl phenylacetate	PubChem CID: 91694682
7, 11-Dimethyl-3-methylene-1, 6, 10-dodecatriene	PubChem CID: 10407
Eudesma-4(14),11-diene<br>Synonyms: β-Eudesmene; β-Selinene	PubChem CID: 442393
3-Methyl-2-phenylethyl butanoic acid ester<br>Synonyms: Phenethyl isovalerate	PubChem CID: 8792
α-Farnesene	PubChem CID: 5281516
3, 7-Dimethyl-2,6-octadienyl butanoic acid ester	PubChem CID: 7796
1, 2, 3, 5, 6, 8a-Hexahydro-4, 7-dimethyl-1-(1-methylethyl)-naphthalene	No results
1, 4-Diethyl-1, 4-dimethyl-2, 5-cyclohexadiene	No results
1H-Indole-3-butanoic acid	PubChem CID: 8617
6-Isopropenyl-3-methyl-cyclohex-1-enol	No results
2-(4a-methyl-8-methylene-decahydro-naphthalene-2-yl)-propan-2-ol	No results
Tricyclo [4.3.1.0(3, 8)] decan-10-ol	PubChem CID: 584172
2,2-Dimethyl-propionic acid 5-isopropenyl-2-methyl-cyclohex-2-enyl ester	No results

Allyl phenoxyacetate	PubChem CID: 24117	
5,5-Dimethyl-8-methylene-1,2-epoxycyclooct-3-ene	No results	
6-isopropenyl-3-methyl-cyclohex-2-enol	No results	
2-pentacosanone	PubChem CID: 547856	
(Z, Z)-9,12-octadeca-dienoic acid	No results	
E-9-Tetradecenoic acid	PubChem CID: 5312402	
Pentatriacontane	PubChem CID: 12413	
(z)-3-Heptadecen-5-yne	PubChem SID: 273541236	
Tetratetracontane	PubChem CID: 23494	
α-Cadien	No results	Extraction method: GC-MS<br>Reference: [23]
Asaron	PubChem CID: 636822	
Carveol	PubChem CID: 7438	
Cnidiadin	PubChem CID: 101937463	Extraction method: HPLC-MS<br>Reference: [24]
Diosmetin	PubChem CID: 5281612	Extraction method: HPLC-MS-ESI-UV<br>Reference: [25]
Dl-umtatin	No results	
Cnidimol C	PubChem CID: 70697378	
Cnidimol E	No results	
5-Formylxanthotoxol	PubChem SID: 272958592	
Edultin	PubChem CID: 5317013	
(3' R)-3'-Hydroxy-columbianedin	No results on PubChem but chemical structure presented in previous review	Extraction method: HPLC-ESI-MS

		Reference: [26]
3,7-Dimethyl-3(E)-octene-1,2,6,7-tetraol	No results	Extraction method: HPLC-[1] HNMR<br><br>Reference: [27]
L-camphene	PubChem CID: 440966	Extraction method: N/A<br><br>Reference: [28]
Bornyl isovalerate	PubChem CID: 60968	
2-Butene	PubChem CID: 62695	Extraction method: GC-MS<br><br>Reference: [29]
2-Methylacrolein	PubChem CID: 6562	
Toluol	PubChem CID: 1140	
Isobutyric acid-isopropyl ester	PubChem CID: 12044	
2-Ethylidene-1,1-dimethylcyclopentane	PubChem CID: 5370115	
2-Methylbutyric Acid Isopropyl Ester	PubChem SID: 87560796	
Pentanoate isopropyl ester<br><br>Synonyms: Isopropyl valerate	ChemSpider: ID79025	
1,7,7-Trimethylbicyclo [2.2.1] heptane	PubChem CID: 92108	
4(10)-Thujene	PubChem CID: 18818	
1,4,8-Menthatriene	PubChem CID: 527141	
P-Cymene	PubChem CID: 7463	
3,5-Dimethylstyrene	PubChem CID: 21476	
1,3,7-Octatriene,3-7-dimethyl	PubChem CID: 5320249	
1,5,8-Menthatriene<br><br>Synonyms: P-menth-1,5,8-triene	PubChem CID: 527424	
2,2,3-Trimethyl-(R)-3-cyclopentene-1-acetaldehyde	PubChem SID: 134984169	

Heptane,1-methyl-4-(1-methylethenyl)-7-Oxa bicyclo [4,1,0]	PubChem CID: 91496	
Myrtenol	PubChem CID: 10582	
Dihydrocarvone	PubChem CID: 24473	
Verbenone	PubChem CID: 29025	
Trans-carveol	PubChem CID: 94221	
Carvol	PubChem CID: 16724	
Pinocamphone	PubChem CID: 11038	
Isopiperitenone	PubChem CID: 79036	
Fructus perillae aldehyde	No results	
Z-methyl geranate	PubChem CID: 5365912	
Geranyl acetate	PubChem CID: 1549026	
Benzyl isovalerate	PubChem CID: 7651	
Phenethyl isobutyrate<br>Synonyms: Propionic acid-2-methyl-2-phenyl ethyl ester	PubChem CID: 7655	
Decahydro-2α-methyl-6-methylene-1-(1-met hylethyl)<br>cyclobutyl [1,2,3,4] dicyclopentenyl	No results	
Trans-caryophyllene<br>Synonyms: β-Caryophyllene; Caryophyllene	PubChem CID: 5281515	
Geranyl isobutyrate	PubChem CID: 6086514	
β-Eudesmol	PubChem CID: 91457	Extraction method: N/A<br>Reference: [30]
Cyclofenchene	PubChem CID: 79022	Extraction method: N/A

β-Terpinene	PubChem CID: 66841	Reference: [31]
Isoborneol<br><br>Synonyms: Borneol	PubChem CID: 64685	
Azulene	PubChem CID: 9231	
1(7),8(10)-p-menthadien-9-ol	PubChem CID: 34627	
Dipentene oxide	PubChem CID: 232703	
α-Cubebene	PubChem CID: 86609	
α-Bergamotene	PubChem CID: 86608	
α-Elemene	PubChem CID: 80048	
Dimethyl ketene	PubChem CID: 136395	Extraction method: GC-MS<br><br>Reference: [32]
Undecane	PubChem CID: 14257	
Cis-carveol	PubChem CID: 330573	
7H-furo[3,2-g] [1] benzopyran-7-one,9- [(4-hydroxy-<br><br>3-methyl-2-buten 1) oxy]-[E]	No results	
Nerolidol	PubChem CID: 5284507	
Nerolidol isomer	PubChem CID: 5320128	
Cis-isopropenyl-2-methylene-3-cyclohexyl-acetate	No results	
Propionate	PubChem CID: 7336	
Tert-butyl phenyl acetate	PubChem CID: 316203	
Diisobutyl phthalate	PubChem CID: 6782	
Linoleic acid	PubChem CID: 5280450	
Oleic acid	PubChem CID: 445639	

Synonyms: 9-Octadecanoic acid		
Stearic acid<br>Synonyms: Octadecanoic acid	PubChem CID: 5281	
Arachidic acid<br>Synonyms: Eicosanoic acid	PubChem CID: 10467	
Ethyl arachidate	PubChem CID: 29009	
Propionic-2-methyl-1-methylethyl ester	No results	Extraction method: GC-MS<br>Reference: [33]
Nonane	PubChem CID: 8141	
α-Methylbenzyl ethanol	PubChem SID: 223742051	
Trans-sabinene hydrate	PubChem CID: 12315151	
Hydroxycitronellal	PubChem CID: 7888	
1-Methyl-4-(1-methylethenyl) cyclohexene	PubChem CID: 439250	
β-Terpineol	PubChem CID: 8748	
α-Pineneoxide	PubChem CID: 91508	
Vanillin	PubChem CID: 1183	
4-(1-methylethyl)-benzyl alcohol	No results	
3-Cyclohexene-1-methanol, α, α,4-trimethyl	PubChem CID: 17100	
2-Butyl-1-methyl-pyrrolidine	PubChem CID: 558513	
Pentanoate-1,3,3-trimethyl-bicyclo [2,2,1] hept-<br>2-yl-ester	No results	
1-Methyl-3-(1-methylethyl)-phenyl	No results	
2-Methyl-5-(1-methylethyl)-2-cyclohexen-1-ketone	No results	

β-Dihydrofuran	Patent ID: US9212174	
1,7,7-Trimethyl-bicyclo [2.2,1] heptane-2-ol acetate	No results	
14-Hydroxycaryophyllene	PubChem CID: 5352484	
Trans-carvacryl acetate	No results	
α-Muurolene	PubChem CID: 12306047	
Cubenol	PubChem CID: 519857	
Trans-dihydro thuja alcohol	No results	
Bicyclogermacrene	PubChem CID: 13894537	
Dihydrocalamenene	No results	
6-Methyl-α-ionone	PubChem CID: 5371002	
Trans sesquihydrate Sabinene	No results	
Widdrol	PubChem CID: 94334	
4-α-Hydroxy-dihydro-furan-agar	No results	
α-Bisabolol	PubChem CID: 1549992	
2-Camphanyl angelic acid ester	No results	
Methyl jasmonate	PubChem CID: 5281929	
Furfuryl heptadec-one	No results	
Cis,cis-farnesol	PubChem CID: 1549107	
3-Methyl-2-butene-1-ol	PubChem CID: 11173	Extraction method: GC-MS<br>Reference: [34]
2-Pentyl-furan	PubChem CID: 19602	
Dehydro-p-cymene	PubChem CID: 62385	
Nonanal	PubChem CID: 31289	

β-Linalool<br>Synonyms: Linalool; 3,7-Dimethyl-1,6-octadiene-3-ol; 3,7-Dimethyl-1,6-octadiene-3-ol	PubChem CID: 6549
Fenchol	PubChem CID: 15406
2,6-Dimethyl-1,3,5,7-octatetraene	PubChem CID: 5368451
α-Terpinenol	PubChem CID: 443162
Thymol methyl ether	PubChem CID: 14104
Undecanal	PubChem CID: 8186
Ylangene	PubChem CID: 6432119
β-Elemene	PubChem CID: 6918391
Dodecanal	PubChem CID: 8194
Isocaryophyllene	PubChem CID: 5281522
α-Cedrene	PubChem CID: 11106485
α-Santalene	PubChem CID: 94164
Thujopsene	PubChem CID: 442402
α-Caryophyllene	PubChem CID: 5281520
Acoradiene	PubChem CID: 90351
β-Chamigrene	PubChem CID: 442353
γ-Muurolene	PubChem CID: 12313020
2-Tridecanone	PubChem CID: 11622
β-Himachalene	PubChem CID: 15095
ar-Curcumene	PubChem CID: 92139
γ-Elemene	PubChem CID: 6432312

α-Cadinene	PubChem CID: 12306048	
α-Longipinene	PubChem CID: 520957	
α-Himachalene	PubChem CID: 11830551	
Germacrene B	PubChem CID: 5281519	
Perillyl acetate	PubChem CID: 61780	
Geranyl propionate	PubChem CID: 5355853	
Geranyl 3-methyl-butyrate	No results	
Myristic acid<br>Synonyms: Tetradecanoic acid	PubChem CID: 11005	
Hexahydro farnesyl acetate	No results	
3,7,11,15-Tetramethyl-2-hexadecen-1-ol	PubChem CID: 5366244	
Palmitoleic acid	PubChem CID: 445638	
2-Methyl-2-β-butane-1-ol	No results	Extraction method: GC-MS<br>Reference: [35]
Isopropyl-isobutyric acid	No results	
α-Sung terpene	No results	
2(10)-Sung terpene	No results	
Pentylbenzene	PubChem CID: 10864	
Bicyclo [2.2.1] heptan-2-ol,1,3,3-trimethyl-,2-acetate	PubChem CID: 61696	
5-Propene-2-cyclopenten-1-ol	No results	
2-Methyl-5-propene-2-cyclopentene-1-acetate	No results	
1-Phenyl-1-pentanone	PubChem CID: 66093	
1-Propyl-3,4-dimethoxybenzene	No results	

α-Selinene	PubChem CID: 10856614	
Cinnamyl acetate	PubChem CID: 5282110	
Cedrol	PubChem CID: 65575	
8-(3-methyl-2-β) hem	No results	
P-mentha-E-2,8(9)-dien-1-ol	No results	Extraction method: GC-MS<br>Reference: [21]
3-Cyclohexaen-1-ol<br>Synonyms: 3-Cyclohexen-1-ol	PubChem CID: 556685	
4-Methyl-l-(1-methylethyl)-3-cyclohexaen-1-ol	No results	
Octadecanal	PubChem CID: 12533	
1-Octadecene	PubChem CID: 8217	
Stigmast-5-en-3-ol (3beta, 24s)<br>Synonyms: Clionasterol; γ-Sitosterol	PubChem CID: 457801	
6,6-Dimethyl-2-bicyclo [3,1,1] heptane	No results	Extraction method: GC-MS<br>Reference: [22]
4-Methyl-l-(1-methylethyl)-2-cyclohexene-1-ol	No results	
5-Isopropenyl-2-methyl-cyclohex-2-enyl propionic acid ester	No results	
1,4-Dimethyl-3-cyclohexene-1-ethanol	No results	
Tricyclo [4.4.0.0 (2,8)] decan-4-ol	PubChem CID: 564289	
5-Isopropenyl-3-methyl-cyclohex-1-enol	No results	
Liposoluble compounds		
2,4-Dimethylhexane	PubChem CID: 11511	Extraction method: GC-MS<br>Reference: [36]
2,3-Dimethylhexane	PubChem CID: 11447	

2,3,4-Trimethylhexane	PubChem CID: 13533	(with voucher specimen)
2-Methylheptane	PubChem CID: 11594	
3-Methylheptane	PubChem CID: 11519	
Octane	PubChem CID: 356	
5-Ethyl-2,4-dimethyl-2-heptene	PubChem CID: 557846	
1,2,5,5-Tetramethyl-1,3-cyclopentadiene	PubChem CID: 572142	
Heptadecane	PubChem CID: 12398	
Z-11-tetradecenoic acid	PubChem CID: 5362743	
Octadecane	PubChem CID: 11635	
Pentadecanoic acid	PubChem CID: 13849	
Nonadecane	PubChem CID: 12401	
(z)-7-Hexadecenoic acid	PubChem CID: 5318393	
Eicosane	PubChem CID: 8222	
Heptadecanoic acid	PubChem CID: 10465	
6,9,12-Octadecatrienoic acid	PubChem CID: 3453	
9,12-Octadecadienoic acid	PubChem CID: 3931	
9,12,15-Octadecatrienoic acid	PubChem CID: 860	
Phytol	PubChem CID: 5280435	
7,10,13-Eicosatrienoic acid	PubChem CID: 5312532	
Heneicosane	PubChem CID: 12403	
Docosanoic acid	PubChem CID: 8215	
Tricosanoic acid	PubChem CID: 17085	

Tetracosanoic acid	PubChem CID: 11197	
Nonacosane	PubChem CID: 12409	
Stigmast-4-en-3-one	PubChem CID: 5484202	
Hexacosanoic acid	PubChem CID: 10469	
Tocopherols	PubChem CID: 14986	
Octacosanoic acid	PubChem CID: 10470	
Chromones		
Cnidimol B	ChemSpider ID:10235600	Extraction method: ESI-MS<br>Reference: [37]<br>(with voucher specimen)
Peucenin	PubChem CID: 68477	
5,7-Dihydroxychromone	PubChem CID: 5281343	
5-O-Methylvisamminol	PubChem CID: 441970	
4′-O-β-D-glucosyl-5-O-methylvisamminol	PubChem CID: 21670038	
Hamaudol	PubChem CID: 164722	
2,5-Dimethyl-7-hydroxychromone	PubChem CID: 5316891	
Cimifugin	PubChem CID: 441960	
5-Hydroxy-chromone-7-O-β-D-glucoside	No results	
Eduotin IV	No results	Extraction method: HPLC-MS<br>Reference: [24]
Oroselone	PubChem CID: 74477	Extraction method: PCEC<br>Reference: [20]
Cnidimol A	PubChem CID: 129317384	Extraction method: N/A<br>Reference: [12]

Cindimol F	No results	Extraction method: ^{1}H+^{13}C-NMR<br>Reference: [38]
Cindimoside A	No results	Extraction method: HPLC-GC-MS<br>Reference: [14]<br>(Voucher number: C-2103)
Hydroxycnidimoside A	PubChem CID: 70689051	Extraction method: ^{1}H+^{13}C-NMR<br>IR + UV spectroscopy<br>SHPLC + CC + TLC<br>Reference: [15]
Undulatoside A	PubChem CID: 5321494	Extraction method: ^{1}H+^{13}C-NMR<br>Reference: [39]
Saikochromoside A	PubChem CID: 70697379	
Cnidimoside B	PubChem CID: 70682745	
2-Methyl-5-hydroxy-6-(2-butenyl-3-hydroxy methyl)-7-(β-D-glucopyranosyloxy)-4H-1-benzopyran-4-one	No results	
Monnieriside A	PubChem CID: 70686986	
Monnieriside B	PubChem CID: 70695364	
Monnieriside C	PubChem CID: 70686988	
Monnieriside D	PubChem CID: 70682747	
Monnieriside E	PubChem CID: 70689052	
Monnieriside F	PubChem CID: 70682748	
Monnieriside G	PubChem CID: 124510520	
6′-Hydroxylangelicain	PubChem CID: 70695365	

Monoterpenoid glucosides		
3,7-Dimethyloct-1-ene-3,6,7-triol 3-O-β-D-glucopyranoside	PubChem CID: 10641593	Extraction method: ^{13}C-NMR<br>Reference: [40]
Enzymatic hydrolysis	No results	
(2s)-3,7-Dimethyloct-3(10),6-diene-1,2-diol 2-O-β-D-glucopyranoside	No results	
(4S)-P-menth-1-ene-7,8-diol 8-O-β-D-glucopyranoside	No results	
(3′R)-Hydroxymarmesin 4′-O-β-D-glucopyranoside<br>Synonyms: Smyrindioloside	PubChem CID: 10836072	
Xanthotoxol 8-O-β-D-glucopyranoside	No results	
2-Methyl-5,7-dihydroxychromone 7-O-β-D-glucopyranoside	No results	
3,7-Dimethyloctane-1,2,6,7-tetrol	PubChem CID: 10655888	Extraction method: ^{13}C-NMR<br>Reference: [41]
(6,7-threo)-3,7-Dimethyloct-3(10)-ene-1,2,6,7,8-pentol	No results	
(6,7-erythro)-3,7-Dimethyloct-3(10)-ene-1,2,6,7,8-pentol	No results	
3,7-Dimethyl-1,2,6,7-tetrahydroxy-oct-3(10)-ene	No results	Extraction method: ^{1}H+^{13}C-NMR<br>Reference: [42]
1-Triacetate	PubChem CID: 219721	
3-Methyl-1,2,3,4-tetrahydroxy-butane	No results	
2-Triacetate	No results	
3,7-Dimethyl-3β,8-dihydroxy-oct-1,6-diene 3-O-β-D-glucopyranoside	No results	
Glycerol	PubChem CID: 753	
Terpenoids, Glycosides, Glucides		

(2S,3R)-2-Methylbutane-1,2,3,4-tetrol<br>Synonyms: 2-C-methyl-D-erythritol	PubChem CID: 11400799	Extraction method: ^{1}H+^{13}C-NMR<br>Reference: [42]
Lactone	PubChem CID: 5585	Extraction method: IR + UV spectroscopy<br>Reference: [43]
Alloimperatorin	PubChem CID: 69502	
Cnidimin	PubChem CID: 91698393	
Cnidioside A	Chemspider ID: 10213905	Extraction method: N/A<br>Reference: [44]
Cnidioside B	No results	
Cnidioside C	No results	
Cnidiol B	No results	
Cnidiol C	PubChem CID: 26396	
3,7-Dimethyloct-1-ene-3,6,7-triol	ChemSpider ID: 10260607	Extraction method: ^{13}C-NMR<br>Reference: [41]
Trans-p-menthane-1β,2α,8,9-tetrol	No results	
Torilin	PubChem CID: 6450226	Extraction method: MS-^{1}D NMR-^{2}D NMR<br>Reference: [45]<br>(Voucher number: WP194)
Torilolone	PubChem CID: 10444938	
1-Hydroxyhorilin	No results	
Picraquassioside A	PubChem CID: 85502992	Extraction method: SGCC-SPHPLC<br>Reference: [46]
Methylpicraquassioside A	PubChem CID: 85363137	
Picraquassioside B	No results	
Methylpicraquassioside B	No results	
Xanthotoxol-8-β-glucoside	No results	
5-Methoxy-xanthotoxol-8-β-glucoside	No results	
8-Methoxy-xanthotoxol-5-β-glucoside	No results	

Marmesinin	PubChem CID: 216283	
L-rhamnose	PubChem CID: 25310	
D-xylose	PubChem CID: 135191	Extraction method: N/A<br>Reference: [47]
L-arabinose	PubChem CID: 439195	
D-galactose	PubChem CID: 6036	
D-mannose	PubChem CID: 18950	
D-apiose	ChemSpider ID: 16735670	
Erythritol	PubChem CID: 222285	Extraction method: ^{13}C-NMR<br>Reference: [40]
D-threitol	PubChem CID: 169019	
D-mannitol	PubChem CID: 6251	
2-Deoxy-d-ribono-1,4-lactone	PubChem CID: 161815	Extraction method: HPLC<br>Reference: [48]<br>(Voucher number: MU961715Y)
Glycerol-2-O-α-L-fucose galactoside	No results	
6-Deoxy-D-glucitol<br>Synonyms: D-quinovitol	PubChem CID: 151266	
1-Deoxy-D-glucitol	PubChem CID: 3429	
D-hamamelose	PubChem CID: 193393	
Other compounds		
Palmitic acid<br>Synonyms: N-hexadecanoic acid; Hexadecanoic acid	PubChem CID: 985	Extraction method: N/A<br>Reference: [49]
Sitosterol<br>Synonyms: Beta-sitosterol 22,23-dihydro-stigmasterol; β-Sitosterol	PubChem CID: 222284	
P-coumaric acid	PubChem CID: 637542	Extraction method: N/A

		Reference: [12]
Thymine<br>Synonyms: 5-Methyl uracil	PubChem CID: 1135	Extraction method: N/A<br>Reference: [50]
Hypoxanthine	PubChem CID: 135398638	
Uracil	PubChem CID: 1174	
L- (+) valine	PubChem CID: 6287	
D-Phenylalanine	PubChem CID: 71567	
Cnideoside A	No results	Extraction method: N/A<br>Reference: [51]
Cnideoside B	No results	

CEC: Capillary Electro-Chromatography

HPLC: High Performance Liquid Chromatography

GC-MS: Gas chromatography–mass spectrometry

UHPLC-ESI-Q-TOF-MS: Ultra-high-performance liquid chromatography coupled with electrospray ionization quadrupole time-of flight tandem mass spectrometry

2D-CCC-HPLC: two-dimensional counter current chromatography-high performance liquid chromatography

HPLC–ESI-MS: high performance liquid chromatography–electrospray ionization-mass spectrometry

HFCF + HPLC: Hollow fiber cell fishing with high performance liquid chromatography

HPLC-PDA-ESI-ITMS^n/TOF-MS: high-performance liquid chromatography coupled with photodiode array, electrospray ionisation ion trap tandem mass spectrometry and time of flight mass spectrometry

UPLC/ESI-TOFMS/MS: ultra-performance liquid chromatographic coupled to electrospray ionization time of flight mass

TLC-MS: Thin layer chromatography/mass spectrometry

^{1}H+^{13}C-NMR,

^{1}H+^{13}C-Nuclear magnetic resonance,

IR + UV spectroscopy: Infared spectra + Ultraviolet spectra

HPLC + CC + TLC: high performance liquid chromatography + column chromatography + Thin layer chromatography

ESI + HRESI: Electrospray ionisation and high-resolution electrospray ionization-mass spectra

CC + TLC + PTLC: column chromatography + Thin layer chromatography + preparative thin-layer chromatography

PCEC: pressurized capillary electrochromatography

SGCC-SPHPLC: silica gel column chromatography + semi-preparative high-performance liquid chromatography

References

1. Chen, Z., et al., *A fast and accurate method for the pharmacokinetic research of four coumarin analogs in Fructus cnidii using capillary electro-chromatography with a methacrylate ester-based monolithic column.* Electrophoresis, 2017. **38**(22-23): p. 3036-3047.

2. Song, L., Jiao., et al., *Simultaneous determination of coumarins and fingerprint analysis in Cnidium monnieri(L.) Cuss.* Tianjin Journal of Traditional Chinese Medicine, 2016(06).

3. Song, G.S., et al., *Simultaneous quantification of 7 coumarins in common cnidium fruit by GC-MS.* Yaoxue Xuebao, 2016. **51**(4): p. 626-630.

4. Jia, M., et al., *Qualitative Analysis and Quality Evaluation of Cnidium monnieri Using UHPLC-ESI-Q-TOF/MS.* Chinese Herbal Medicines, 2016. **8**(4): p. 323-330.

5. Wang, D., et al., *On-line two-dimensional countercurrent chromatography×high performance liquid chromatography system with a novel fragmentary dilution and turbulent mixing interface for preparation of coumarins from Cnidium monnieri.* Journal of Chromatography A, 2015. **1406**: p. 215-223.

6. Song, G., et al., *Simultaneous quantification of 16 bioactive constituents in Common cnidium fruit by liquid chromatography-electrospray ionization-mass spectrometry.* Journal of Pharmaceutical and Biomedical Analysis, 2015. **107**: p. 304-310.

7. Xue, X., et al., *Hollow fiber cell fishing with high performance liquid chromatography for screening bioactive compounds from traditional Chinese medicines.* J Chromatogr A, 2013. **1280**: p. 75-83.

8. Gao, F., et al., *Optimal extraction and fingerprint analysis of Cnidii fructus by accelerated solvent extraction and high performance liquid chromatographic analysis with photodiode array and mass spectrometry detections.* Food Chem, 2013. **141**(3): p. 1962-71.

9. Dien, P.H., et al., *Main constituents from the seeds of Vietnamese Cnidium monnieri and cytotoxic activity.* Nat Prod Res, 2012. **26**(22): p. 2107-11.

10. Li, K., Ping., C. Gao, and W. Li, Min., *Analysis of coumarins in extract of Cnidium monnieri by ultra-performance liquid chromatographic coupled to electrospray ionization time of flight mass/mass spectrometry.* Chinese Traditional Patent Medicine, 2009(04).

11. Liu, S.S., F. Li, and W. Zheng, *Medicine Research Abstracts*, in *Science Press*. 1979, Science Press: Bei Jing. p. 1962–1974.

12. Baba, K., et al., *Chemical studies on Chinese crude drug 'She Chuang Zi'.* Shoyakugaku Zasshi, 1985. **39**: p. 282–290.

13. Liu, J.Q., et al., *TLC-MS identification of coumarins from extracts of Cnidium monnieri (L.) Cusson.* J. Instrumental Anal, 1999. **18**: p. 26–28.

14. Cai, J.N., et al., *Coumarins from the fruits of Cnidium monnieri.* J. Nat. Prod, 2000. **63**: p. 485–488.

15. zhao, J., Yu., et al., *Chromones and coumarins from the dried fructus of Cnidium monnieri.* Fitoterapia, 2011. **82**: p. 767–771.

16. Chang, C.-I., et al., *8-Alkylcoumarins from the fruits of Cnidium monnieri protect against hydrogen peroxide induced oxidative stress damage.* Int. J. Mol. Sci., 2014. **15**: p. 4608–4618.

17. Lee, T.-H., et al., *New Coumarins and Anti-Inflammatory Constituents from the Fruits of Cnidium monnieri.* Int. J. Mol. Sci., 2014(15): p. 9566–9578.

18. Duan, X., Hong., et al., *Chemical constituents isolated from fruits of Cnidium monnieri and their effects on proliferation of UMR106 cells.* Chinese Traditional and Herbal Drugs, 2016(17).

19. Chen, Q., et al., *Identification and quantification of the volatile constituents in Cnidium monnieri using supercritical fluid extraction followed by GC-MS.* Journal of Separation Science, 2009. **32**(2): p. 252-257.

20. Chen, D., et al., *Separation and determination of coumarins in Fructus cnidii extracts by pressurized capillary electrochromatography using a packed column with a monolithic outlet frit.* J Pharm Biomed Anal, 2009. **50**(5): p. 695-702.

21. Zhu, Y., Y. Gu, Hua., and L. Zhu, *Analysis of the Essential Oil from Cnidium monnieri in Two Different Producing Areas by GC-MS.* China Pharmacy, 2008(33).

22. Zhao, F., Chun., et al., *The Volatile Constitutes of Cnidium Monnieri by GC/MS.* Journal of Chinese Mass Spectrometry Society, 2008(06).

23. Zhao, L.H., et al., *Studies on fingerprints of Fructus cnidii by GC and GC-MS.* Chinese Pharmaceutical Journal, 2007. **42**(12): p. 889-891.

24. Yang, X., S.-P. Yang, and X. Zhang, *[Chromatographic fingerprint of Cnidium monnieri].* Yao xue xue bao = Acta pharmaceutica Sinica, 2007. **42**(8): p. 877-881.

25. Chen, Y., et al., *Fingerprint analysis of the fruits of Cnidium monnieri extract by high-performance liquid chromatography-diode array detection-electrospray ionization tandem mass spectrometry.* Journal of Pharmaceutical and Biomedical Analysis, 2007. **43**(3): p. 926-936.

26. Jiang, Y.Q., *Analysis of coumarins in Fructus Cnidii by HPLC-ESI-MS.* Zhong yao cai = Zhongyaocai = Journal of Chinese medicinal materials, 2006. **29**(10): p. 1033-1035.

27. Osorio, C., et al., *Stereochemistry of (3E)-3,7-dimethyl-3-octene-1,2,6,7-tetraol isolated from Passiflora quadrangularis.* Tetrahedron Asymmetry, 1999. **10**(22): p. 4313-4319.

28. Nakao, M., *About Cnidium ingredients.* Pharmaceut. J., 1920: p. 685.

29. Meng, Z.M., C.G. Jin, and S.X. Zhao, *The GC-MS analysis of volatile oil of fructus Cnidii.* J. Nanjing Coll. Pharm, 1986. **17**: p. 167.

30. Xiang, R.D., et al., *Primary studies on chemical constituents of the volatile oil of Cnidium monnieri (L.) Cusson.* J. Chin. Pharm. Univ, 1989. **20**: p. 92–93.

31. Qin, L.P., H. Wu, and T.J. Wang, *Component analysis of volatile oil in fruit of Cnidium monnieri and Cnidium dahuricum.* Chin. Tradit. Herb. Drugs, 1992. **23**: p. 330.

32. Wang, H.B., et al., *Applied study of supercritical-CO2 fluid extraction in extracting volatile constituents of Cnidium monnieri seeds.* China journal of Chinese materia medica, 1996. **2**: p. 018.

33. Qiu, Q., et al., *Determination of chemical constituents of the essential oil from Cnidium monnieri by GC-MS. .* J. Chin. Med. Mater, 2002. **25**: p. 561.

34. Zhou, Y., hong., Y. Li, lin., and L. Wang, sheng. , *Analysis of chemical components of the essential oil from Fructus Cnidii.* Guangxi Univ. Nat. Sci. Edit, 2005. **30**: p. 263.

35. Zhu, Y., *Determination of chemical constituents of the essential oil from Cnidium by GC-MS.* Li Shi Zhen Med. Mater. Med. Res, 2006. **10**: p. 055.

36. Ji, Z., Qiang., et al., *Analysis of Liposoluble Constituents in Fruit of Cnidium Monnieri by GC-MS.* China Pharmacist, 2015(11).

37. Duan, X., Hong., et al., *Chomones from fruit of Cnidium monnieri and their effects on proliferation of UMR106 cells.* Chinese Traditional and Herbal Drugs, 2015(22).

38. Kimiye, B., et al., *Chormones from Cnidium monnieri.* Phytochemistry 1992. **31**: p. 1367–1370.

39. Kim, S., Beom., et al., *Anti-adipogenic chromone glycosides from Cnidium monnieri fruits in 3T3-L1 cells.* Bioorg. Med. Chem. Lett, 2012. **22**: p. 6267–6271.

40. Kitajima, J., et al., *Monoterpenoid glucosides of Cnidium monnieri fruit.* Chemical and Pharmaceutical Bulletin, 1999. **47**(5): p. 639-642.

41. Kitajima, J., et al., *Monoterpenoid polyols in fruit of Cnidium monnieri.* Chemical and Pharmaceutical Bulletin, 1998. **46**(10): p. 1580-1582.

42. Kitajima, J. and Y. Tanaka, *New monoterpenoid and hemiterpenoid tetraol of the crude drug 'She chuang zi'.* Chemical and Pharmaceutical Bulletin, 1993. **41**(9): p. 1667-1669.

43. Nikonov, G.K., *Investigation of lactones of monniers cnidum-cnidium monnieri (L) cuss.* Journal of General Chemistry Ussr, 1964. **34**(4): p. 1350-&.

44. Yahara, S., et al., *Studies on the constituents of Cnidii monnieri Fructus.* J. Pharmacogn, 1993. **47**: p. 74–78.

45. Oh, H., et al., *Sesquiterpenes with hepatoprotective activity from Cnidium monnieri on tacrine-induced cytotoxicity in Hep G2 cells.* Planta Medica, 2002. **68**: p. 748–749.

46. Kim, S., Beom., et al., *A new phenolic glycoside from Cnidium monnieri fruits.* Nat. Prod. Res., 2013. **27**: p. 1945–1948.

47. Hai, H. and H.P. Zeng, *Research on polysaccharides of Cnidium monnieri.* . Guangzhou Chem. Ind., 1995. **23**: p. 29–30.

48. Kitajima, J., T. Ishikawa, and Y. Aoki, *Glucides of Cnidium monnieri fruit.* Phytochemistry, 2001. **58**: p. 641–644.

49. Xiang, R.D. and X.H. Fu, *Study of the chemical constituents of Cnidium(I).* . Chin. Tradit. Herb. Drugs 1984. **9**: p. 14.

50. Xiang, R.D., *Research on active constituents of water extracts of Cnidium monnieri.* . Chin. Tradit. Herb. , 1999. **11**: p. 813.

51. Chen, Y., G. Zhang, Gang., and Z. Yu, Ping., *The advancement in the chemical and pharmacological study of the fruits of Cnidium monnieri.* Journal of Shenyang Pharmaceutical University, 2006. **23**(4).
